# Acute retinal necrosis in a patient with cervical malignant tumor treated with sintilimab: a case report and literature review

**DOI:** 10.3389/fimmu.2024.1301329

**Published:** 2024-01-23

**Authors:** Pei Wang, Ming An, Mengmeng Zhang, Xiaoran Yan, Nianting Tong

**Affiliations:** ^1^ Department of Ophthalmology, Qingdao Hospital, University of Health and Rehabilitation Sciences (Qingdao Municipal Hospital), Qingdao, Shandong, China; ^2^ School of Medicine, Qingdao University, Qingdao, Shandong, China; ^3^ Department of Pathology, Qingdao Hospital, University of Health and Rehabilitation Sciences (Qingdao Municipal Hospital), Qingdao, Shandong, China

**Keywords:** acute retinal necrosis, immune checkpoint inhibitor (ICI), sintilimab, cervical malignant tumor, varicella-zoster virus (VZV)

## Abstract

Acute retinal necrosis (ARN) is an inflammatory disease that is primarily caused by herpesvirus infection, most commonly varicella-zoster virus (VZV), followed by herpes simplex virus (HSV) and occasionally cytomegalovirus (CMV). Sintilimab is an immune checkpoint inhibitor (ICI) that can enhance the body’s anti-tumor immune response. However, treatment with ICIs may lead to reactivation of the VZV. Here, we present a case of ARN caused by VZV infection in a patient receiving sintilimab for cervical cancer. A 64-year-old female patient developed vision loss and floaters with left eye redness for one week after 22 cycles of sintilimab for cervical cancer. Based on clinical manifestations, ophthalmological examination, and vitreous humor biopsy, the patient was diagnosed with acute retinal necrosis syndrome secondary to VZV. After receiving systemic antiviral and anti-inflammatory therapy, retinal necrosis lesions and visual function improved. In conclusion, clinicians should be aware of the risk of ARN when using sintilimab and should actively monitor patients for prompt diagnosis and optimal management of this rare adverse drug reaction.

## Introduction

Acute retinal necrosis (ARN) is a rare syndrome characterized by acute panuveitis with periretinal inflammation, diffuse necrotizing retinitis, and retinal detachment (1). The incidence of ARN is not clear, but two national studies in the United Kingdom estimated an annual incidence of 0.5-0.63 cases per million population (2). The herpes virus family, including varicella-zoster virus (VZV), herpes simplex virus (HSV), cytomegalovirus (CMV), and Epstein-Barr virus (EBV) (3), has been identified as the primary causative pathogens of ARN, although human adenovirus (HAdV) (4) and pseudorabies-virus (PRV) (5) have also been reported to cause ARN.

Immunotherapy, including immune checkpoint inhibitors (ICIs), has become an important part of systemic anticancer treatment (6). However, it has been reported that such new drugs can cause some negative effects due to their unique mechanism of action, such as immunotoxicity, hyperprogression, and reactivation of certain diseases, etc (7).

This report describes a case of ARN secondary to VZV during immunotherapy with sintilimab injection for cervical cancer. It is thought that treatment with ICIs may lead to reactivation of the VZV (8, 9). As far as we know, no such adverse reaction has been reported previously.

## Case presentation

**Figure 1:**
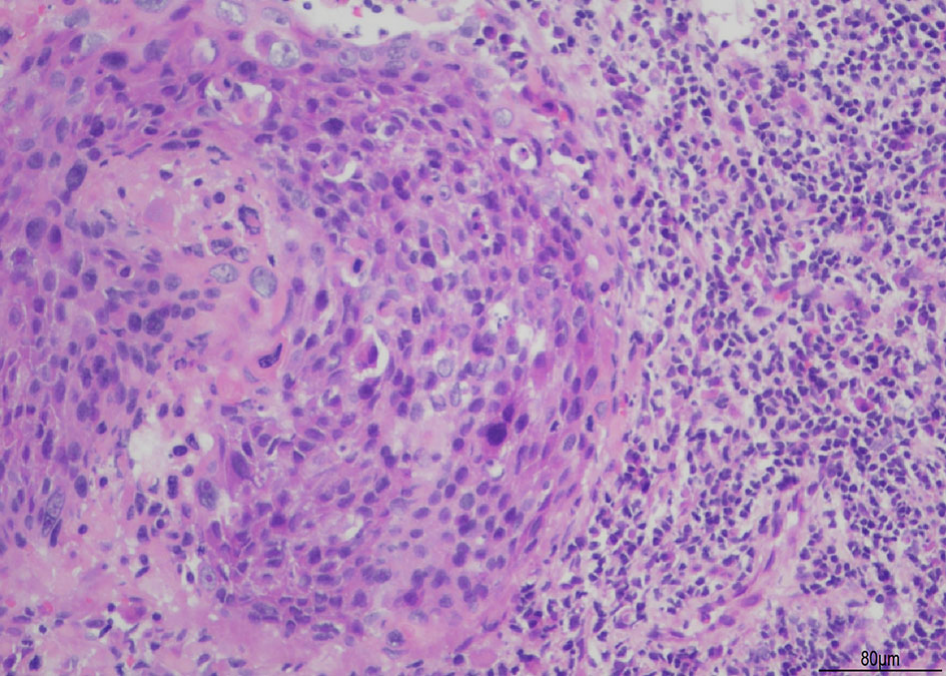
illustrates squamous cell carcinoma of the cervix. The histological section is stained with Hematoxylin and Eosin (H&E). The images are captured at an original magnification of X200, revealing round or oval-shaped cancer cells with irregularly round or oval nuclei that exhibit deep staining. There is an increased ratio of nuclear to cytoplasmic volume, and pathological mitosis is observed. In addition, a significant infiltration of inflammatory cells can be observed surrounding the cancerous mass.

**Figure 2:**
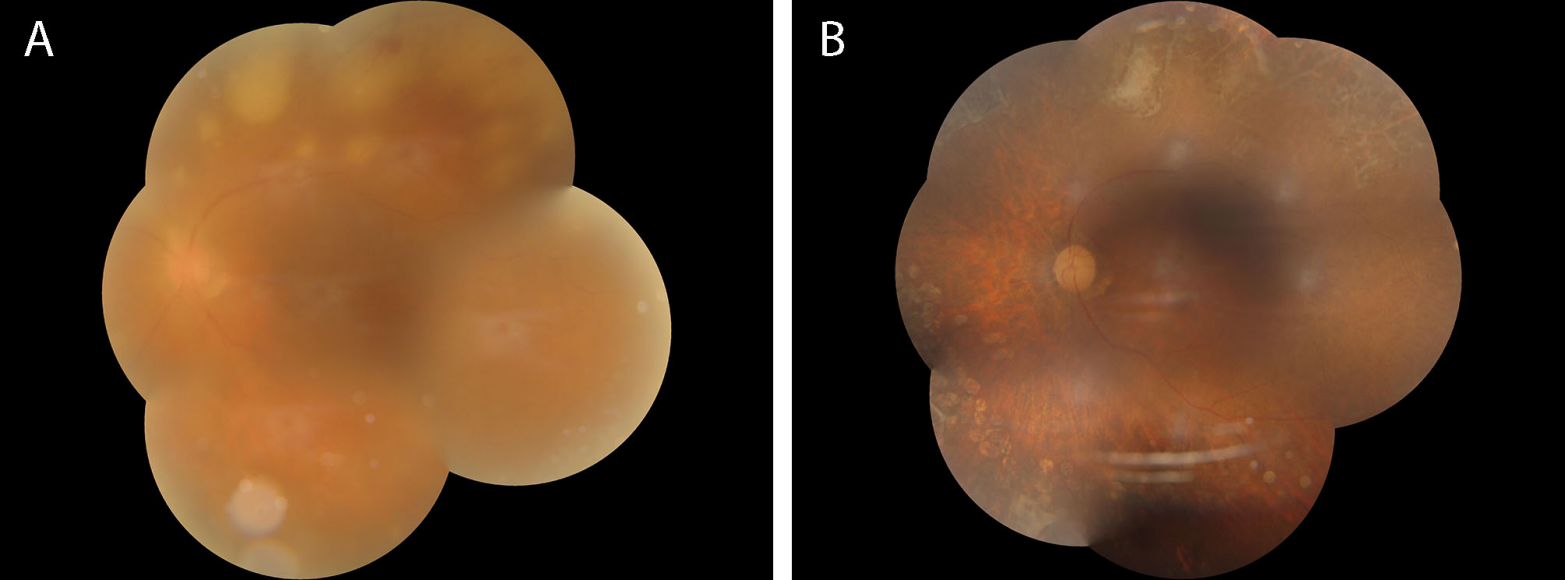
**(A)** shows the fundus photograph of the patient during their first visit, indicating severe opacity in the vitreous body, extremely thin retinal arteries, and multiple yellow-white necrotic lesions with clear boundaries of varying sizes visible in the peripheral retina outside the vascular arch. Partially fused necrotic lesions can be seen inside the necrotic lesions, accompanied by flake-shaped bleeding. **(B)** shows the fundus photograph of the patient at their last follow-up after 40 weeks of anti-inflammatory and antiviral treatment. The opacity in the vitreous body has disappeared, and the retinal arteries appear as white lines. The previously yellow-white necrotic lesions have disappeared, leaving multiple retinal atrophic lesions with pigmentary disturbances remaining.

A 64-year-old female patient was admitted to the ophthalmic department of our hospital due to decreased vision and floaters accompanied by redness of the left eye for 1 week. The patient had been diagnosed with stage IIB squamous cell carcinoma of the cervix 4 years ago (**Figure 1**). Starting in 2018, the patient underwent two cycles of chemotherapy using lobaplatin in conjunction with paclitaxel. A review in February 2019 revealed an increase in tumor size, prompting the administration of six cycles of chemotherapy involving nedaplatin combined with tegafur-gimeracil-oteracil potassium capsules (S-1), along with two cycles of external radiation therapy. Upon reassessment, the cervical lesions of the patient had nearly disappeared. Subsequently, the patient underwent five cycles of irinotecan combined with cisplatin chemotherapy and received two cycles of external radiotherapy for consolidation treatment. Following this, in a tumor-free state from 2021 to the present, the patient has undergone 22 cycles of sintilimab immune maintenance therapy. At presentation, the best corrected visual acuity was 20/80 and the intraocular pressure was 18mmHg. The left eye had many keratic precipitates (KPs), positive anterior chamber cells, and moderate to severe vitreous clouding. Ophthalmoscopy of the posterior pole showed scattered deep gray-white retinal lesions and local fusion in the equatorial and peripheral retinas (**Figure 2A**).

After testing vitreous fluid samples, PCR analysis revealed varicella-zoster virus 5.01 × 10^7^ copies/ml, while IL-6 and IL-8 were also significantly increased to 18,811.2pg/ml and 9579.0pg/ml, respectively, supporting the diagnosis of active ARN.

After definitive diagnosis, the patient underwent standard antiviral and anti-inflammatory treatments, including local treatment with intravitreal injection of ganciclovir (2.5 mg/0.1 ml) twice weekly for 4 consecutive weeks and intravenous injection of ganciclovir (0.25 g) twice daily for 2 weeks, followed by oral valacyclovir (0.3 g) three times daily for 20 weeks. Additionally, topical ganciclovir gel was applied for antiviral therapy, while tobramycin and low-dose dexamethasone eye drops were used for anti-inflammatory therapy. Three days after starting standard systemic antiviral treatment, the patient’s vitreous inflammation was evident and anti-inflammatory treatment was initiated with oral prednisone at a dose of 0.5 mg/kg body weight, gradually tapered by 20% each week until a maintenance dose of 5 mg/day (one tablet) was reached and continued in combination with oral valacyclovir. After 4 weeks of standardized anti-inflammatory and antiviral treatment, the necrotic lesion in the patient’s fundus was controlled without further progression. During the course of ARN treatment, the patient consulted with an oncologist and opted to continue receiving sintilimab. Unfortunately, the patient experienced a recurrence of vitreous opacity and anterior chamber inflammation worsened, resulting in posterior synechiae. Considering the patient’s unstable condition, a standard pars plana vitrectomy (PPV) was performed, with the removal of the cloudy vitreous for histopathological examination again. After vitreous removal, fusion laser photocoagulation was performed around the necrotic lesion in the retina. The vitreous biopsy showed a significant decrease in VZV viral copy number to 3.93 × 10^4^ copies/ml compared to before, while IL-6 and IL-8 were also significantly decreased to 10,182.9pg/ml and 2221.8pg/ml, respectively, indicating that the previous antiviral and anti-inflammatory treatments were effective. The patient continued to receive antiviral and anti-inflammatory treatments after surgery, and the disease remained stable. After 20 weeks of systemic antiviral treatment, the patient discontinued antiviral medication and oral prednisone. At the last follow-up 40 weeks after onset, the corrected visual acuity remained stable at 20/80, with the resolution of the necrotic lesion in the fundus (**Figure 2B**), residual pigment disturbance, and visible retinal atrophy holes around the upper necrotic lesion, which were well-closed by laser photocoagulation.

## Discussion

ARN is a syndrome usually caused by the herpes virus family (10). The differential diagnosis of this entity includes other causes of infectious uveitis, such as progressive outer retinal necrosis (PORN) (11), CMV retinitis (12), toxoplasmosis (13), and syphilis, as well as non-infectious conditions like intraocular lymphoma (14) and Behcet’s disease (15). PORN mainly affects HIV-positive patients with varicella-zoster virus infection, and there is some etiology overlap with ARN. However, it has multiple very rapid fusion lesions, showing only small vitreous infiltration, and mild typical retinal vasculitis, starting from the posterior pole. In contrast, CMV retinitis begins to conceal along the retinal vessels, usually showing extensive retinal hemorrhage. The retinal changes of toxoplasmosis are more focal than ARN, showing less concomitant infiltration, and are responsive to anti-toxoplasmosis drugs. At present, polymerase chain reaction (PCR) of aqueous humor or vitreous and vitrectomy biopsy are effective methods for the diagnosis of ARN and the best way to guide its antiviral treatment (16). Early diagnosis and identification of the type of virus infection can guide the use of antiviral drugs and improve the visual prognosis of patients (17).

Typically, ARN occurs in immunocompetent individuals; however, it may also occur in immunocompromised patients, such as those who are suppressed via corticosteroids (18), non-corticosteroid immunosuppressants (19), and chemotherapeutics (20). Viral reactivation is considered the main route of infection in ARN in infected cases. Emotional stress, trauma, surgery, and other stress can induce the activation of herpesvirus from the ganglion back to the peripheral tissue to enter a new round of replication infection (21). Our present case of ARN was caused by VZV during immunotherapy with sintilimab. It is speculated that it is also related to the reactivation mechanism of the virus.

Sintilimab belongs to ICI, which can bind to programmed death-ligand 1 (PD-L1), to reduce the inhibitory effect of the tumor on T cells and stimulate anti-tumor immunity (22). Previous clinical trial data have shown that sintilimab may be efficacious and safe as a second-line and later treatment for patients with advanced cervical cancer with disease progression after chemotherapy (23). However, with the widespread use of immunotherapy, its adverse events have gradually been discovered. These include immune-related adverse events (irAEs), toxicity of immunotherapy combined with other drugs, reactivation of diseases such as tuberculosis (TB) and nodular granulomatosis, tumor hyperprogression (HPD), and other more lethal outcomes (7). Up to now, ARN has not been previously reported in relation to sintilimab or any other ICI, for that matter.

Regarding the causes of ARN in patients receiving sintilimab, it may related to ICI-induced VZV reactivation. Firstly, immune reconstitution inflammatory syndrome (IRIS) caused by ICI may increase the incidence of VZV (9). Secondly, activated T lymphocytes infiltrate non-cancerous tissues and increase existing autoantibodies and inflammatory cytokines. The perturbation of self-tolerance by ICI therapy may lead to reactivation (24, 25). Moreover, we suggest that sintilimab could interfere with the binding of PD-L1 and PD-1 on T lymphocytes, which could eliminate the self-tolerance and immune privilege of organs like the eyes, resulting in eye inflammation (26). Finally, we believe that the cause of ARN may be related to the patient’s own physical condition. Despite using sintilimab injection to up-regulate T lymphocytes, the patient’s immunity remains low due to a malignant cervical tumor, resulting in insufficient overall lymphocytes and viral reactivation, leading to ARN development.

The treatment of ARN involves drug therapy, laser therapy, and surgical treatment. Initial antiviral therapy includes intravenous acyclovir or oral valaciclovir, and other systemic drugs like famciclovir and valganciclovir, and intravenous foscarnet or ganciclovir (27). Ganciclovir or valganciclovir is typically used to treat CMV retinitis, with ganciclovir replacing acyclovir if the etiological test is VZV, or acyclovir has not shown significant improvement (28). Most patients receive topical steroids during initial treatment, while some advocate oral corticosteroids 24 to 48 hours after antiviral therapy starts to minimize the risk of vitreous inflammation and retinal detachment. Preventive retinal laser photocoagulation may reduce retinal detachment (29), but there is no convincing evidence for its use (30). It remains controversial whether early vitrectomy should be performed to prevent retinal detachment (10, 31).

In our present case, throughout the entire course of treatment, the patient had been receiving treatment with sintilimab. During the first month of standardized antiviral and anti-inflammatory treatment for ARN, the patient experienced recurrent vitreous opacity and intraocular inflammation, and we could not rule out whether these relapses were related to the use of sintilimab. However, in subsequent treatment, we performed vitrectomy and intraoperative prophylactic laser therapy, and the postoperative vitreous biopsy showed a significant decrease in viral copies and inflammatory factors, indicating that the overall treatment was still effective. Therefore, we continued with the previous treatment plan, and after 6 weeks of treatment, the patient’s intraocular inflammation gradually stabilized and remained stable until the last follow-up. Reflecting on the patient’s entire treatment process, we found that standardized antiviral and anti-inflammatory treatment may be effective for ARN patients who require continuous use of sintilimab due to their overall condition. However, we need to closely monitor the patient’s eye condition and take timely interventions such as surgery and laser therapy when inflammation relapses. At the same time, intraocular fluid biopsy during treatment was very important, because it can provide us with valuable information to determine treatment efficacy and guide our subsequent treatment plan.

In conclusion, ophthalmic adverse reactions should not be overlooked in patients with malignant tumors when using sintilimab. A comprehensive evaluation of ophthalmic risk factors and a detailed inquiry into ophthalmic history should be conducted before using the drug. During and after treatment, continuous follow-up of the patient’s eye conditions is crucial, and relevant ophthalmic examinations should be regularly performed if necessary.

## Data availability statement

The raw data supporting the conclusions of this article will be made available by the authors, without undue reservation.

## Ethics statement

Written informed consent was obtained from the individual(s) for the publication of any potentially identifiable images or data included in this article.

## Author contributions

PW: Data curation, Formal analysis, Investigation, Methodology, Writing – original draft. MA: Data curation, Investigation, Methodology, Supervision, Writing – original draft. MZ: Data curation, Investigation, Writing – original draft. XY: Data curation, Methodology, Writing – review & editing. NT: Conceptualization, Data curation, Funding acquisition, Investigation, Methodology, Supervision, Validation, Writing – original draft, Writing – review & editing.
